# Supplementary material for: Kinetic and Mechanistic Study of Polycarbodiimide Formation from 4,4′-Methylenediphenyl Diisocyanate
Source: Int J Mol Sci. 2025 Sep 3;26(17):8570. doi: 10.3390/ijms26178570 (PMC12429836; doi:10.3390/ijms26178570)
Supplement: Supplementary file 1 [file ijms-26-08570-s001.zip › ijms-3802586-supplementary.pdf]

Kinetic and Mechanistic Study of Polycarbodiimide Formation from 4,4′-Methylenediphenyl Diisocyanate

Marcell D. Csécsi [1,2], R. Zsanett Boros [3], Péter Tóth [3], László Farkas [3] and Béla Viskolcz [1,2,*]

[1] Institute of Chemistry, University of Miskolc, Miskolc-Egyetemváros, H-3515 Miskolc, Hungary; marcell.daniel.csecsi@uni-miskolc.hu
[2] Higher Education and Industrial Cooperation Centre, University of Miskolc, H-3515 Miskolc, Hungary
[3] BorsodChem Ltd., Bolyai tér 1, H-3700 Kazincbarcika, Hungary; renata.boros@borsodchem.eu (R.Z.B.); peter.toth45@borsodchem.eu (P.T.); laszlo.farkas@borsodchem.eu (L.F.)
* Correspondence: bela.viskolcz@uni-miskolc.hu

Supporting Information

Table S1. Data charts of the 50, 60, 70, 80 °C measurements, including CO_2 gas volumes in cm³ ($V_{g,t}$) as raw data by the time (t), and derived data: gas moles according to ideal gas law ($n_{g,t}$), MDI moles from gross equation ($n_{MDI,t}$), conversion (X_t), solution volume ($V_{S,t}$), MDI concentration ($[MDI]_t$) reciprocal MDI concentration for plotting $\left(\frac{1}{2[MDI]_t}\right)$ and PCDI-2 concentration from gross equation ($[PCDI\text{-}2]_t$).

Measurement	Reading time, t [min]	Gas volume, $V_{g,t}$ [cm³]	Gas moles, $n_{g,t}$ [mol]	Reacted MDI, $n_{MDI,t}$ [mmol]	MDI conversion, X_t [%]	Solution volume, $V_{S,t}$ [cm³]	MDI concentration, $[MDI]_t$ [mol dm⁻³]	Reciprocal concentration, $\frac{1}{2[MDI]_t}$ [mol⁻¹ dm³]	PCDI-2 concentration, $[PCDI\text{-}2]_t$ [mol dm⁻³]
50 °C	0.0	0.0	0.000000	0.0000	0.00	15.6709	0.5151	0.9707	0.00000
	0.5	0.6	0.000024	0.0488	0.60	15.6701	0.5120	0.9766	0.00156
	1.0	1.7	0.000069	0.1383	1.71	15.6686	0.5063	0.9875	0.00441
	1.5	3.2	0.000130	0.2603	3.22	15.6665	0.4986	1.0028	0.00831
	2.0	4.8	0.000195	0.3904	4.84	15.6642	0.4904	1.0196	0.01246
	2.5	6.7	0.000273	0.5450	6.75	15.6616	0.4806	1.0404	0.01740
	3.0	8.7	0.000354	0.7077	8.77	15.6588	0.4703	1.0632	0.02260
	3.5	10.4	0.000423	0.8460	10.48	15.6564	0.4615	1.0833	0.02702
	4.0	12.2	0.000496	0.9924	12.29	15.6539	0.4523	1.1056	0.03170
	4.5	13.6	0.000553	1.1063	13.71	15.6519	0.4450	1.1235	0.03534
	5.0	15.6	0.000634	1.2690	15.72	15.6491	0.4347	1.1502	0.04054
	6.0	18.7	0.000761	1.5211	18.84	15.6447	0.4187	1.1941	0.04861
	7.0	21.2	0.000862	1.7245	21.36	15.6412	0.4058	1.2321	0.05513
	8.0	24.2	0.000984	1.9685	24.39	15.6370	0.3903	1.2810	0.06294
	9.0	26.8	0.001090	2.1800	27.01	15.6334	0.3769	1.3267	0.06972
	10.0	29.2	0.001188	2.3752	29.43	15.6300	0.3645	1.3718	0.07598
	12.0	33.5	0.001363	2.7250	33.76	15.6240	0.3422	1.4610	0.08721
	14.0	37.8	0.001535	3.0707 *	38.04 *	15.6180	0.3202 *	1.5614 *	0.09831 *
	16.0	42.2	0.001714	3.4281 *	42.47 *	15.6119	0.2975 *	1.6809 *	0.10979 *
	18.0	46.0	0.001868	3.7368 *	46.29 *	15.6066	0.2778 *	1.8000 *	0.11972 *
	20.0	48.6	0.001974	3.9480 *	48.91 *	15.6029	0.2643 *	1.8918 *	0.12652 *
	25.0	52.2	0.002120	4.2405 *	52.53 *	15.5979	0.2456 *	2.0355 *	0.13593 *
	30.0	54.3	0.002206	4.4111 *	54.65 *	15.5950	0.2347 *	2.1300 *	0.14143 *
	35.0	56.7	0.002303	4.6060 *	57.06 *	15.5916	0.2223 *	2.2493 *	0.14771 *
	45.0	57.7	0.002344	4.6873 *	58.07 *	15.5902	0.2171 *	2.3031 *	0.15033 *
	60.0	58.1	0.002360	4.7198 *	58.47 *	15.5896	0.2150 *	2.3253 *	0.15137 *
60 °C	0.0	0.0	0.000000	0	0	15.5846	0.5087	0.9830	0.00000
	0.5	2.2	0.000089	0.1786	2.25	15.5815	0.4973	1.0054	0.00573
	1.0	4.4	0.000179	0.3572	4.51	15.5784	0.4859	1.0290	0.01146
	1.5	6.8	0.000276	0.5520	6.96	15.5751	0.4735	1.0559	0.01772
	2.0	9.4	0.000382	0.7631	9.63	15.5714	0.4601	1.0868	0.02450
	2.5	12.6	0.000511	1.0229	12.90	15.5670	0.4435	1.1273	0.03285

	3.0	15.4	0.000625	1.2502	15.77	15.5630	0.4290	1.1654	0.04017
	3.5	18.6	0.000755	1.5100	19.05	15.5586	0.4125	1.2122	0.04853
	4.0	20.8	0.000844	1.6886	21.30	15.5555	0.4011	1.2467	0.05428
	4.5	23.6	0.000958	1.9159	24.17	15.5516	0.3865	1.2935	0.06160
	5.0	25.6	0.001039	2.0782	26.22	15.5488	0.3762	1.3292	0.06683
	5.5	28.2	0.001145	2.2893	28.88	15.5451	0.3627	1.3786	0.07363
	6.0	30.4	0.001234	2.4679	31.13	15.5421	0.3513	1.4235	0.07939
	6.5	32.4	0.001315	2.6303	33.18	15.5393	0.3409	1.4668	0.08463
	7.0	34.8	0.001413	2.8251	35.64	15.5359	0.3284	1.5225	0.09092
	7.5	36.8	0.001494	2.9875	37.69	15.5331	0.3180	1.5723	0.09616
	8.0	39.4	0.001599	3.1985	40.35	15.5295	0.3045	1.6421	0.10298
	8.5	42.0	0.001705	3.4096 *	43.01 *	15.5258	0.2910 *	1.7184 *	0.10980 *
	9.0	43.6	0.001770	3.5395 *	44.65 *	15.5236	0.2826 *	1.7690 *	0.11400 *
	9.5	45.0	0.001827	3.6531 *	46.08 *	15.5216	0.2754 *	1.8158 *	0.11768 *
	10.0	46.8	0.001900	3.7993 *	47.93 *	15.5191	0.2660 *	1.8798 *	0.12241 *
	11.0	49.6	0.002013	4.0266 *	50.79 *	15.5152	0.2514 *	1.9888 *	0.12976 *
	12.0	53.4	0.002168	4.3351 *	54.69 *	15.5099	0.2316 *	2.1589 *	0.13975 *
	13.0	56.0	0.002273	4.5461 *	57.35 *	15.5063	0.2180 *	2.2931 *	0.14659 *
	14.0	58.6	0.002379	4.7572 *	60.01 *	15.5026	0.2045 *	2.4452 *	0.15343 *
	15.0	61.4	0.002492	4.9845 *	62.88 *	15.4987	0.1899 *	2.6334 *	0.16080 *
	16.0	63.6	0.002582	5.1631 *	65.13 *	15.4956	0.1784 *	2.8030 *	0.16660 *
	17.0	66.6	0.002703	5.4066 *	68.20 *	15.4914	0.1627 *	3.0730 *	0.17450 *
	18.0	68.4	0.002776	5.5528 *	70.05 *	15.4889	0.1533 *	3.2616 *	0.17925 *
	19.0	70.4	0.002858	5.7151 *	72.10 *	15.4861	0.1428 *	3.5004 *	0.18452 *
	20.0	72.6	0.002947	5.8937 *	74.35 *	15.4830	0.1313 *	3.8071 *	0.19033 *
	22.5	77.6	0.003150	6.2996 *	79.47 *	15.4760	0.1052 *	4.7544 *	0.20353 *
	25.0	81.2	0.003287	6.5744 *	82.93 *	15.4713	0.0874 *	5.7182 *	0.21247 *
	30.0	86.2	0.003490	6.9792 *	88.04 *	15.4643	0.0613 *	8.1563 *	0.22565 *
	35.0	88.4	0.003579	7.1573 *	90.29 *	15.4613	0.0498 *	10.0415 *	0.23146 *
	40.0	91.0	0.003684	7.3678 *	92.94 *	15.4576	0.0362 *	13.8172 *	0.23832 *
	45.0	91.4	0.003700	7.4002 *	93.35 *	15.4571	0.0341 *	14.6658 *	0.23938 *
	50.0	92.4	0.003741	7.4812 *	94.37 *	15.4557	0.0289 *	17.3266 *	0.24202 *
	55.0	92.8	0.003757	7.5136 *	94.78 *	15.4551	0.0268 *	18.6825 *	0.24308 *
	60.0	93.4	0.003781	7.5621 *	95.40 *	15.4543	0.0236 *	21.1676 *	0.24466 *
70 °C	0.0	0.0	0.000000	0.0000	0.00	15.6678	0.5119	0.9767	0.00000
	0.5	3.3	0.000134	0.2674	3.33	15.6632	0.4950	1.0101	0.00853
	1.0	9.0	0.000365	0.7292	9.09	15.6553	0.4658	1.0735	0.02329
	1.5	14.6	0.000591	1.1829	14.75	15.6474	0.4370	1.1442	0.03780
	2.0	19.4	0.000786	1.5718	19.60	15.6407	0.4123	1.2126	0.05025
	2.5	24.2	0.000980	1.9607	24.44	15.6340	0.3876	1.2899	0.06270
	3.0	29.6	0.001199	2.3982	29.90	15.6265	0.3598	1.3896	0.07673
	3.5	34.6	0.001402	2.8033	34.95	15.6195	0.3340	1.4968	0.08974
	4.0	39.4	0.001596	3.1921	39.80	15.6128	0.3093	1.6167	0.10223
	4.5	44.4	0.001799	3.5972	44.85	15.6058	0.2835	1.7640	0.11525
	5.0	48.8	0.001975	3.9498 *	49.24 *	15.5998	0.2610 *	1.9160 *	0.12660 *
	5.5	53.0	0.002145	4.2897 *	53.48 *	15.5939	0.2393 *	2.0897 *	0.13754 *
	6.0	56.9	0.002303	4.6054 *	57.42 *	15.5885	0.2191 *	2.2821 *	0.14772 *
	7.0	64.0	0.002590	5.1800 *	64.58 *	15.5786	0.1823 *	2.7420 *	0.16626 *
	8.0	71.0	0.002873	5.7466 *	71.65 *	15.5688	0.1461 *	3.4230 *	0.18456 *
	9.0	77.3	0.003128	6.2565 *	78.00 *	15.5600	0.1134 *	4.4098 *	0.20104 *
	10.0	83.4	0.003375	6.7502 *	84.16 *	15.5515	0.0817 *	6.1201 *	0.21703 *
	12.5	93.8	0.003792	7.5845 *	94.56 *	15.5372	0.0281 *	17.8044 *	0.24407 *
	15.0	103.0	0.004164	8.3283 *	103.83 *	15.5243	-	-	0.26824 *
80 °C	0.0	0.0	0.000000	0.0000	0.00	15.5810	0.5114	0.9776	0.00000
	0.5	5.0	0.000203	0.4054	5.09	15.5741	0.4856	1.0296	0.01301
	1.0	12.0	0.000486	0.9729	12.21	15.5643	0.4495	1.1124	0.03125
	1.5	18.4	0.000746	1.4917	18.72	15.5553	0.4164	1.2008	0.04795
	2.0	26.6	0.001078	2.1565	27.06	15.5439	0.3739	1.3372	0.06937
	2.5	33.0	0.001338	2.6754	33.57	15.5350	0.3407	1.4674	0.08611
	3.0	39.0	0.001581	3.1618	39.68	15.5266	0.3096	1.6150	0.10182
	3.5	45.4	0.001840	3.6807	46.19	15.5176	0.2763	1.8094	0.11860
	4.0	51.0	0.002067	4.1347 *	51.89 *	15.5098	0.2472 *	2.0227 *	0.13329 *

4.5	55.6	0.002254	4.5076 *	56.57 *	15.5034	0.2232 *	2.2397 *	0.14538 *
5.0	60.4	0.002448	4.8968 *	61.45 *	15.4967	0.1982 *	2.5223 *	0.15800 *
5.5	65.2	0.002643	5.2859 *	66.33 *	15.4900	0.1732 *	2.8869 *	0.17062 *
6.0	69.6	0.002821	5.6427 *	70.81 *	15.4838	0.1502 *	3.3283 *	0.18221 *
6.5	73.4	0.002975	5.9507 *	74.68 *	15.4785	0.1304 *	3.8351 *	0.19223 *
7.0	77.2	0.003129	6.2588 *	78.54 *	15.4732	0.1105 *	4.5245 *	0.20225 *
7.5	81.8	0.003314	6.6273 *	83.17 *	15.4669	0.0867 *	5.7652 *	0.21424 *
8.0	84.2	0.003411	6.8218 *	85.61 *	15.4635	0.0742 *	6.7412 *	0.22058 *
8.5	88.6	0.003589	7.1783 *	90.08 *	15.4574	0.0511 *	9.7774 *	0.23220 *
9.0	91.0	0.003686	7.3727 *	92.52 *	15.4540	0.0386 *	12.9644 *	0.23854 *
9.5	93.6	0.003792	7.5834 *	95.16 *	15.4504	0.0249 *	20.0462 *	0.24541 *
10.0	96.2	0.003897	7.7940 *	97.81 *	15.4468	0.0113 *	44.2040 *	0.25229 *
10.5	98.4	0.003986	7.9723 *	100.04 *	15.4437	-	-	0.25811 *
11.0	101.0	0.004091	8.1829 *	102.69 *	15.4401	-	-	0.26499 *
11.5	103.2	0.004181	8.3612 *	104.92 *	15.4370	-	-	0.27082 *
12.0	105.4	0.004270	8.5394 *	107.16 *	15.4339	-	-	0.27664 *
12.5	107.4	0.004348	8.6957 *	109.12 *	15.4312	-	-	0.28175 *

*These values are over the limit of applicability, thus not accurate, because in the calculation it was assumed that 1 mole of carbon dioxide is always produced from 2 moles of MDI reactant. Due to derived reacted MDI, conversion and reciprocal concentration values are overestimated, while unreacted MDI and concentration are underestimated after the initial stage of the reaction, where stoichiometric calculation is already not applicable.

Table S2. Raw thermochemical data of theoretical calculations at B3LYP/6-31G(d)[SMD(ODCB)] level of theory, extracted from optimized Gaussian output files (2nd-4th columns) and calculated relative energies (5th column). These are calculated with the formula: $\Delta E^{‡} = 2625.5 \cdot [E_{TS} - (E_{MDI} + E_{MPPO})]$ (applying Hartree → kilojoule conversion). The energy highlighted with bold refers to the rate determining activation enthalpy barrier of the reaction. MDI—4,4'methylene diphenyl diisocyanate, MPPO—3-methyl-1-phenyl-2-phospholene-l-oxide catalyst, TS—transition state of the rate determining step.

	MPPO (Hartree)	MDI (Hartree)	TS (Hartree)	Relative TS energies
Zero-point correction =	0.213753	0.216548	0.430655	–
Thermal correction to Energy =	0.226078	0.232559	0.460034	–
Thermal correction to Enthalpy =	0.227022	0.233503	0.460978	–
Thermal correction to Gibbs Free Energy =	0.174199	0.168432	0.364385	–
Sum of electronic and zero-point Energies =	−843.399518	−837.386307	−1680.765781	52.6255 kJ mol^{-1}
Sum of electronic and thermal Energies =	−843.387194	−837.370296	−1680.736402	55.3665 kJ mol^{-1}
Sum of electronic and thermal Enthalpies =	−843.386250	−837.369352	−1680.735458	**52.8881 kJ mol^{-1}**
Sum of electronic and thermal Free Energies =	−843.439073	−837.434423	−1680.832051	108.8138 kJ mol^{-1}
Entropy (cal mol^{-1} K^{-1}) =	111.177	136.953	203.297	−187.581 J mol^{-1} K^{-1}
Heat capacity (C_v, cal mol^{-1} K^{-1}) =	47.44	59.777	110.756	14.807 J mol^{-1} K^{-1}

Table S3. Cartesian coordinates of the optimized structures, computed in Gaussian with B3LYP/6-31G(d) level of theory at 298.15 K and 1 atm in solvent phase, applying SMD implicit model for orthodichlorobenzene. MDI—4,4'-methylenediphenyl diisocyanate, MPPO—3-methyl-1-phenyl-2-phospholene-l-oxide catalyst, TS—transition state of the rate determining step.

MDI	MPPO	TS
N 4.72013400 -0.93497100 0.67815500	P -0.46869600 0.91197800 -0.08999500	P 2.85889200 -0.55897600 0.50278200
C 3.57983500 -0.19915500 0.33512400	O -0.37864100 2.35665200 -0.52640000	O 4.71725700 1.75835900 1.12512800
C 3.32254800 0.19647300 -0.98372800	C -1.32794600 0.57517800 1.51631700	C 3.97709200 -1.15370000 1.83968600
C 2.68073300 0.14455700 1.35306200	C -2.66693200 -0.10608000 1.15482700	C 3.20485400 -2.27904800 2.56827700
C 2.17160000 0.92936500 -1.27087400	C -2.62661600 -0.57122800 -0.29064300	C 1.99096100 -2.68097700 1.75575600
C 1.53614200 0.87547400 1.04804700	C -1.56771900 -0.17089200 -1.01837200	C 1.69928800 -1.91720900 0.68539700
C 1.26050600 1.28193900 -0.26639100	C 1.18996200 0.13726300 -0.03992700	C 3.68891900 -0.69036300 -1.12279400
C 5.70511300 -1.42604800 0.18407400	C -3.75949100 -1.41095200 -0.80160000	C 1.19132400 -3.86950200 2.19698900
H 4.02029700 -0.06905600 -1.77296200	C 1.36382000 -1.23540100 0.19676100	C 3.74512300 0.42187700 -1.97319000
H 2.88861600 -0.16410200 2.37294800	C 2.31505500 0.94663300 -0.24644800	C 4.26299200 -1.90505500 -1.53167300

H 1.98301200 1.23527300 -2.29707800	C 2.64525100 -1.78667500 0.23093600	C 4.37004800 0.32160300 -3.21815700
H 0.84485400 1.13285900 1.84655100	C 3.59745200 0.39385500 -0.21250000	C 4.88836400 -2.00077400 -2.77439900
C 0.01084300 2.08688700 -0.58903700	C 3.76341200 -0.97201900 0.02697400	C 4.94262200 -0.88720600 -3.61838700
H 0.01227800 3.01208800 0.00000200	H -1.46138200 1.51556300 2.05912900	H 4.23436900 -0.31630200 2.48857500
H 0.05079300 2.38680600 -1.64313100	H -0.70363100 -0.07905400 2.13236500	H 4.90141300 -1.52564900 1.38880000
C -1.29010000 1.34584000 -0.32328700	H -3.51269700 0.58321100 1.28176600	H 2.87251500 -1.94745700 3.56071400
C -2.17655400 1.77607100 0.67125900	H -2.86960900 -0.95715300 1.81718700	H 3.84928600 -3.15011200 2.73852100
C -1.63635100 0.20853000 -1.07105300	H -1.42989500 -0.40806400 -2.06997800	H 0.85033200 -2.08116800 0.03075000
C -3.37132100 1.09966700 0.92011400	H -3.83566500 -2.34588600 -0.23016100	H 1.81693500 -4.77193900 2.20188600
H -1.93391500 2.65676100 1.26069000	H -3.64172700 -1.65855700 -1.86061500	H 0.32679500 -4.04557000 1.55086700
C -2.82396200 -0.47699800 -0.83798500	H -4.71576800 -0.88658900 -0.67015900	H 0.83636000 -3.72902700 3.22664600
H -0.96517600 -0.14816300 -1.84850400	H 0.49893000 -1.87655600 0.34841200	H 3.29801900 1.36177300 -1.66424400
C -3.69836300 -0.03082300 0.16585200	H 2.17577400 2.00676500 -0.43699400	H 4.21903100 -2.77672200 -0.88381400
H -4.05285100 1.44045800 1.69336700	H 2.77204800 -2.85056100 0.41342800	H 4.40950600 1.18827900 -3.87216600
H -3.08099700 -1.35408200 -1.42558300	H 4.46474800 1.02867600 -0.37388300	H 5.33098700 -2.94321000 -3.08492700
C -5.55832400 -1.63719600 0.08611700	H 4.76094300 -1.40276100 0.05266800	H 5.42967300 -0.96387700 -4.58670900
O -6.29885200 -2.52458400 -0.15569100		C 3.69111100 2.36829500 1.02323300
N -4.90317600 -0.68794400 0.43967600		O 3.09349900 3.39766900 0.98111200
O 6.70389400 -1.95240800 -0.16166800		N 2.27196600 0.93047200 0.81478800
		C 0.95564700 1.32323600 0.51557300
		C 0.24751300 0.90542100 -0.63195800
		C 0.29938900 2.21049800 1.39015000
		C -1.05726800 1.33287400 -0.87056900
		H 0.73033000 0.24830400 -1.35113100
		C -0.99876000 2.64822800 1.13164800
		H 0.82472200 2.55216200 2.27607700
		C -1.70826800 2.21528900 0.00311000
		H -1.57379100 0.97999900 -1.76109900
		H -1.47269500 3.33774300 1.82768600
		C -3.12882300 2.68638300 -0.26781300
		H -3.37898000 3.47438400 0.45362200
		H -3.18300300 3.14754300 -1.26175900
		C -4.17749600 1.58714200 -0.18981400
		C -4.41407400 0.90097200 1.01137700
		C -4.93821100 1.23132800 -1.31172000
		C -5.37518000 -0.10231300 1.09411800
		H -3.83556100 1.15396700 1.89631500
		C -5.90383600 0.22706300 -1.24830100
		H -4.77715100 1.74943000 -2.25413100
		C -6.12460200 -0.44453000 -0.03914500
		H -5.55449700 -0.62624400 2.02802400
		H -6.48540200 -0.03744900 -2.12724000
		N -7.08128700 -1.45971400 0.08016800
		C -7.90451300 -2.05943600 -0.56567200
		O -8.73983400 -2.72229000 -1.07349400

Table S4. Results of IRC calculation (performed with Gaussian) on the rate determining transition state, TS, in the first subprocess, where CO_2 eliminates. Four structures are depicted in Figure 10 in the manuscript.

Number of point on IRC path	Intrinsic Reaction Coordinate	Relative EE (electronic energy) to TS (kJ mol⁻¹)
Stable complex before TS (depicted)	—	−30.82
1 (depicted)	−3.367	−19.43
2	−3.030	−17.41
3	−2.694	−15.02
4	−2.357	−12.34
5	−2.020	−9.58
6	−1.684	−6.93
7	−1.347	−4.57
8	−1.010	−2.63
9	−0.673	−1.18
10	−0.337	−0.29
11 (TS at the local maximum; depicted)	0.000	0.00
12	0.337	−0.26
13	0.674	−0.95
14	1.010	−1.92
15	1.347	−3.10
16	1.684	−4.44
17	2.020	−5.80
18	2.357	−7.19
19	2.694	−8.51
20	3.030	−9.74
21 (depicted)	3.366	−10.90
Stable complex after TS (depicted)	—	−22.06

Table S5. Cartesian coordinates of the optimized, stable complexes before and after the rate determining transition state (TS), AD2 and IM1, respectively, as they were named similarly in our previous publication for phenyl isocyanate. For these calculations, frequency calculations validated the local minima on the potential energy surface. These stable complexes were computed in Gaussian with B3LYP/6-31G(d) level of theory at 298.15 K and 1 atm in solvent phase, applying SMD implicit model for orthodichlorobenzene.

Before TS (AD2)	After TS (IM1)
P 2.87671900 0.52252900 -0.55903000	P -2.97215300 0.83328500 0.24782500
O 3.94202200 -1.01264700 -1.98680800	O -3.69758500 -1.18473400 3.55892800
C 3.09844800 1.65695300 -1.99404500	C -4.02041700 1.66208700 1.51356900
C 2.26859300 2.92603500 -1.71079500	C -3.76437700 3.17972300 1.35930700
C 1.53336900 2.77633300 -0.39530700	C -3.02165700 3.44822000 0.06428800
C 1.73451600 1.63176800 0.28378000	C -2.59718500 2.37010300 -0.62141800
C 4.32577600 0.34942400 0.53225100	C -4.02198800 -0.19582900 -0.85572900
C 0.65384600 3.89989100 0.06425800	C -2.79160300 4.87255200 -0.34455100
C 4.68255500 -0.89243700 1.07380100	C -3.99132600 -1.59189800 -0.72558200
C 5.05719800 1.49546100 0.87486000	C -4.87117400 0.38816900 -1.80921400
C 5.76326800 -0.98386400 1.95132400	C -4.79981500 -2.39239000 -1.53483300
C 6.14516300 1.39606600 1.74392800	C -5.67936100 -0.41418500 -2.61603300
C 6.49768300 0.15745100 2.28423600	C -5.64448000 -1.80477500 -2.47952700
H 2.76057900 1.11836200 -2.88156200	H -3.75158700 1.27954200 2.50001000
H 4.16517400 1.85467300 -2.12332300	H -5.06933800 1.41461600 1.32607400
H 1.54860600 3.11373600 -2.51737400	H -3.16677800 3.56456500 2.19633200
H 2.91220700 3.81406900 -1.66557600	H -4.70676800 3.74095100 1.37542400
H 1.28500800 1.42173600 1.24828400	H -2.02977500 2.42546700 -1.54531900
H 1.23071000 4.83042200 0.14930700	H -3.74852700 5.39459600 -0.47831800
H 0.18816700 3.68715800 1.03073100	H -2.22107600 4.94519700 -1.27495100

H	-0.13876700	4.08927100	-0.67185300	H -2.24778200 5.41331900 0.44174700
H	4.12184000	-1.78359100	0.81140500	H -3.33016200 -2.04926900 0.00440500
H	4.78177700	2.46558000	0.46999000	H -4.89901800 1.46846200 -1.92462300
H	6.03464600	-1.94912300	2.36963600	H -4.76751200 -3.47335000 -1.42869500
H	6.71201800	2.28670000	2.00046500	H -6.33273800 0.04542300 -3.35282700
H	7.34184400	0.08158900	2.96416000	H -6.27244600 -2.42846900 -3.11014600
C	2.89537200	-1.70218200	-1.76764800	C -2.98988500 -2.01582700 3.13916700
O	2.51806700	-2.79171900	-2.18511900	O -2.30725000 -2.88499600 2.75870900
N	2.07124400	-0.93721500	-0.81787700	N -1.79333200 0.02407700 0.97953600
C	0.78450000	-1.32756500	-0.34553300	C -0.64610100 -0.47609400 0.37723500
C	0.50449300	-1.38461900	1.02361800	C -0.35353700 -0.45619700 -1.00694300
C	-0.21112500	-1.69521500	-1.26252900	C 0.33616000 -1.05998200 1.21694100
C	-0.76072200	-1.77708700	1.46679400	C 0.84200300 -0.97757700 -1.50618200
H	1.27831300	-1.13100400	1.74289200	H -1.07305000 -0.03698500 -1.70659800
C	-1.46168900	-2.10533600	-0.80922900	C 1.52177400 -1.57577000 0.70883900
H	0.00885900	-1.66897800	-2.32414100	H 0.13686500 -1.09602800 2.28484800
C	-1.76218900	-2.14697700	0.56161800	C 1.80685600 -1.54662800 -0.66737600
H	-0.96193700	-1.81421400	2.53485900	H 1.02216500 -0.94428500 -2.57963100
H	-2.22178100	-2.38909300	-1.53341200	H 2.24589200 -2.01041100 1.39598400
C	-3.13652400	-2.58349100	1.04509200	C 3.10538600 -2.11298900 -1.21816100
H	-3.38350000	-3.55889800	0.60953000	H 3.21842600 -3.16042900 -0.91048000
H	-3.09728200	-2.72591400	2.13201900	H 3.04562800 -2.11958100 -2.31449600
C	-4.25085800	-1.60469500	0.70687100	C 4.35621600 -1.35592200 -0.79600000
C	-5.29154100	-1.96264200	-0.15865300	C 5.38848500 -1.99636700 -0.09983700
C	-4.26570600	-0.31376400	1.25972400	C 4.51509800 0.00546600 -1.10371400
C	-6.31831200	-1.06940300	-0.46771700	C 6.54504400 -1.31263800 0.27796000
H	-5.30575300	-2.95724800	-0.59728900	H 5.29097600 -3.04980000 0.15112500
C	-5.28171900	0.58873300	0.96412800	C 5.66154000 0.70266600 -0.73768800
H	-3.46814000	-0.00830000	1.93253500	H 3.72574900 0.52978800 -1.63654400
C	-6.31635600	0.21056400	0.09385400	C 6.68552600 0.04058400 -0.04169900
H	-7.12168800	-1.35634600	-1.13913000	H 7.33946900 -1.81869900 0.81790700
H	-5.28292500	1.58360400	1.40055100	H 5.77278200 1.75482300 -0.98521500
N	-7.36013400	1.08472800	-0.23258900	N 7.85780000 0.70026700 0.34729200
C	-7.75313300	2.20181200	-0.00128500	C 8.39113600 1.78062500 0.29201900
O	-8.25083400	3.26411600	0.13337900	O 9.02679900 2.77599500 0.31233600

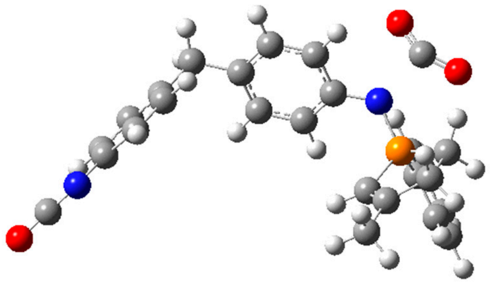

Figure S1. Animation of the rate determining transition state (TS) at the imaginary frequency (−225.95 cm^{-1}), calculated with Gaussian.

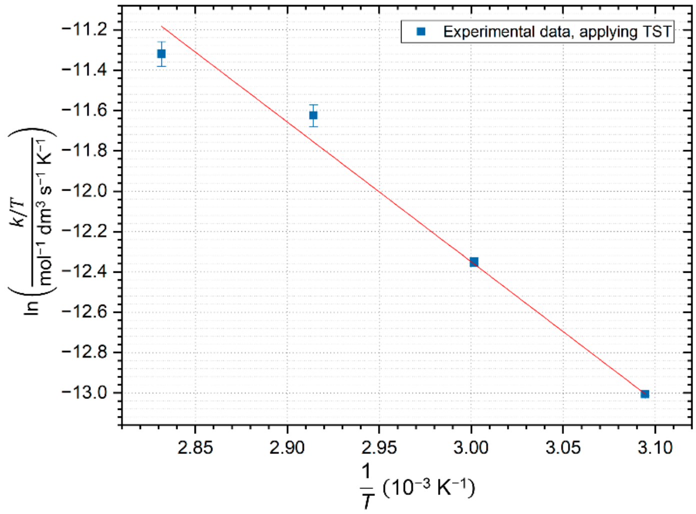

Figure S2. Linearized Eyring plot of rate constants derived from linearized second-order kinetic regression. Fitting parameters give theoretical activation energies: slope gives enthalpy of activation, while intercept gives the entropy of activation, as indicated in the text. Fitted equation: $y = (-6928.56 \pm 364.57)x + (8.43 \pm 1.12)$, $R^2 = 0.9917$.
