# Supplementary figures and images for: Kinetic and Mechanistic Study of Polycarbodiimide Formation from 4,4′-Methylenediphenyl Diisocyanate
Source: Int J Mol Sci. 2025 Sep 3;26(17):8570. doi: 10.3390/ijms26178570 (PMC12429836; doi:10.3390/ijms26178570)

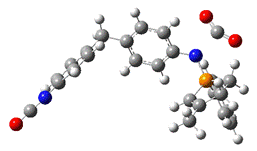

Supplement: Supplementary file 1 [file ijms-26-08570-s001.zip › Figure S1.gif]
